# Early-Life Microbiome and Neurodevelopmental Disorders: A Systematic Review and Meta-Analysis

**DOI:** 10.2174/011570159X360129250508113618

**Published:** 2025-05-26

**Authors:** Md. Fakruddin, Tasbir Amin, Md. Asaduzzaman Shishir, Rameesa Maliha Jameel, Mubashshir Muntaha Bari, Nashia Farzana Shameem, Amana Hossain, Nusrat Jerin, Shahnewaj Bin Mannan, Jinath Sultana Jime, Nayeema Bulbul

**Affiliations:** 1 Department of Biochemistry & Microbiology, North South University, Bashundhara, Dhaka, Bangladesh;; 2 Department of Microbiology, Dhaka International University, Satarkul, Badda, Dhaka, Bangladesh;; 3 H Lee Moffitt Cancer Center and Research Institute 12902 USF Laurel Dr. Tampa, Florida, USA

**Keywords:** Early-life microbiome, neurodevelopmental disorders, gut-brain axis, Autism Spectrum Disorder (ASD), Attention Deficit Hyperactivity Disorder (ADHD), systematic review

## Abstract

**Background and Objectives:**

This systematic review intends to find out how neurodevelopmental disorders, including Attention Deficit Hyperactivity Disorder (ADHD) and Autism Spectrum Disorder (ASD), are influenced by the gut microbiota throughout early childhood. The study looks at the variety and types of microbes that a child is exposed to, the particular microbiome profiles associated with neurodevelopmental outcomes, and the molecular processes that underlie these relationships.

**Methods:**

We performed a thorough search of PubMed, Scopus, the WHO Global Health Library (GHL), and ISI Web of Science. After screening 2,744 original studies based on predetermined eligibility criteria, 19 studies were included. Microbial groupings, presence (high/low), and related neurodevelopmental disorders were among the primary areas of data extraction. The methodological quality of the studies was assessed using the Newcastle-Ottawa Quality Assessment Scale (NOS).

**Results:**

The investigated literature repeatedly showed a strong correlation between dysbiosis of the gut microbiota and neurodevelopmental disorders. Cases of ASD were associated with both a high number of *Clostridium* species and a low number of *Bifidobacterium* species. On the other hand, a Low number of *E. coli* and a high number of the class *Clostridia,* phylum *Firmicute*, genus *Bifidobacterium,* and *Akkermansia,* as well as the species *Listeria monocytogenes*, *Toxoplasma gondii*, *Streptococcus mutans,* and *Mycobacterium tuberculosis* have been linked to ADHD. The NOS evaluation showed variation in the quality of the methodology; some studies had high scores, suggesting sound technique, while other studies had lower scores, indicating serious methodological flaws.

**Conclusion:**

The results highlight the potential impact of the gut microbiome throughout early life on neurodevelopmental outcomes, indicating that microbial imbalances may play a role in the onset of disorders like ASD and ADHD. However, to improve the quality of data, larger-scale longitudinal studies would be required.

## 1 INTRODUCTION

Microorganisms have a vital role in several biological functions within the human body, some of which are essential for our existence [1]. Over a hundred million bacteria, or up to 100 times as many as human cells, live in the human gut and have developed into a mutually advantageous symbiotic condition with the human body over many years of shared evolution. *Firmicutes*, *Bacteroidetes*, *Proteobacteria*, *Actinomycetes*, *Verrucomicrobia*, and *Fusobacteria,* are the six primary phyla that make up these cells; *Bacteroidetes* and *Firmicutes* are the two most prevalent phyla [2]. The gut microbiome is the collective term for these several trillion commensal bacteria that inhabit the human gut [3].

There is a close connection between neurological developmental issues and microorganisms [4]. Neurodevelopmental disorders are a category of neurological illnesses with origins in early brain development, including prenatal and early postnatal stages. They are characterized by deficiencies that range from broad deterioration in socialization and cognitive ability to specific impairments in learning, speech, or motor development [5]. The interplay of genetic, epigenetic, as well as environmental variables determines the multifactorial origin of neurodevelopmental diseases. The clinical variety and phenotypic diversity found in patients are indicative of a complex and diverse etiology, which has complicated the identification of processes leading to their development [6].

Neurodevelopmental disorders (NDs) arise from impairments in the development and function of the brain and nervous system. These disorders typically manifest in early childhood and often persist into adulthood, profoundly impacting cognitive, behavioral, and social functions throughout the lifespan. Among some of the major ones, attention-deficit/hyperactivity disorder (ADHD), autism spectrum disorder (ASD), intellectual difficulties, and communication disorders have been in the light of discussion. Autism Spectrum Disorder (ASD) affects approximately 1% to 2% of the population and is characterized by social impairments, as well as restricted and repetitive behaviors and interests. In contrast, the prevalence of ADHD is 3.4% in adults and 7% in children [7], with serious consequences for mental as well as physical wellness [8]. Early in life, the microbiome forms a symbiotic relationship with the host and goes through a profound developmental process that lasts the duration of the organism. Thus, it was proposed that early life modifications of this microbiome may have an effect on neurodevelopment and could contribute to the possibility of poor neurodevelopmental and mental health outcomes [9].

The effects of gut microbiota on host wellness, as well as physiological processes, including neurodevelopment, have drawn more attention in the past 20 years [10-12]. Several significant biological processes are influenced by the gut microbiota [13]. A growing body of analyses on the subject of the connection between the gut microbiome and the CNS suggests that the gut microbiome could influence CNS functions by means of a pathway known as the microbiota-gut-brain axis (MGBA) [3, 9]. A wide range of bidirectional pathways comprising immunological, hormonal, and neurological signalling make up the MGBA [14]. These microbial metabolites can impact neuroinflammation, neuronal signal transduction, and blood-brain barrier maintenance by activating aryl hydrocarbon receptors (AhR) on astrocytes and microglia, ultimately contributing to a reduction in inflammation [15]. Although AhR is mostly investigated in astrocytes and microglia, the fact that it is present in some neurons raises the possibility that it may have a more extensive regulatory role in the brain, influencing the actions of both neurons and non-neuronal cells in the gut-brain axis [16].

In fact, the gut-brain axis appears to have significance in brain development because it affects the maturation and function of microglia [2]. Among these interactions are microglial reactions to microbial metabolites, which have the ability to alter neuronal signalling, synaptic pruning, and neuroinflammation. In response to signals from the gut microbiota, microglia, the main mediators of neuroinflammation, release proinflammatory cytokines, which affect immunological responses in the central nervous system [17]. This immune activity, which is vitally controlled by resident CNS immune cells as well as gut-derived signals, balances inflammation and neuroprotection during neurodevelopment [18]. Furthermore, because the microbiome modulates immune responses and antigen imitation, it may have a significant impact on the beginning of inflammatory disorders of the central nervous system as well as in the regulation of mucosal and systemic immunological responses [19]. The microbiome may also influence the brain by stimulating peripheral immune cells, which help regulate the body's responses to neuroinflammation, brain injury, autoimmunity, and neurogenesis. These immune cells, which include T-cells and macrophages, react to microbial signals by producing growth factors and cytokines that stimulate neurogenesis, help repair damaged brain tissue, and regulate the activity of microglia in the brain. The relevance of the gut-brain axis in neurodevelopment is highlighted by the indirect effects of the microbiome on immunological responses [20], neuronal health, and brain development through these pathways. Therefore, it is possible that the microbiota plays a role in both *de novo* neurotransmitter synthesis and modulation of neurotransmitter production. Furthermore, the majority of serotonin is located in the gastrointestinal system, indicating a potential function for the microbiota in regulating serotonin synthesis. Tryptophan and other serotonin precursors may be produced by some gut microorganisms, including *Streptococcus*, which helps the gut manufacture serotonin [21]. Additionally, by interacting with enterochromaffin cells, microbial metabolites alter the serotonin pathways of the host and stimulate the release of serotonin, which contributes to gut motility and may have an impact on mood regulation through the gut-brain axis [22]. Additionally, MGBA, norepinephrine, dopamine, and tryptamine have all been linked to microbiota-dependent mechanisms that are particular to neurotransmitter modulation [13]. For example, microbial metabolites such as short-chain fatty acids affect neurotransmitter pathways, including norepinephrine, dopamine, and tryptamine, *via* activating receptors in the gut-brain axis [22]. Tyrosine and tryptophan are neurotransmitter precursors produced by some gut bacteria that aid in the production of dopamine and serotonin. Furthermore, bacteria alter neuroinflammatory processes, which impact the stability and availability of neurotransmitters in the brain *via* modulating aryl hydrocarbon receptors in astrocytes and microglia [23].

According to recent research, gut dysbiosis may be linked to a number of neurological and neurodevelopmental disorders. These studies additionally indicated that the human microbiome ecosystem could be having an impact on behaviour, and central signalling systems, alongside brain development [24, 25]. A correlation has been shown in a few recent studies using animal models between the microbiome and several neuropsychiatric illnesses, including autism spectrum disorders (ASD) and Parkinson's disease [26, 27]. Faecal transplantation has demonstrated some effectiveness in treating children with autism spectrum disorders, as shown by several recent translational researches [28].

Over the past three decades, there has been a major global increase in the body of literature on the incidence of neurological disorders and mental illnesses in children and adolescents. According to a relatively recent meta-analysis, the prevalence of all mental disorders is estimated to be 13.4% globally, making them and their detrimental effects a major public health issue [29]. Regarding the frequency of certain conditions, research by Polanczyk and colleagues [30] indicates that ADHD in children is 3-5% common worldwide, with gender ratios (F: M) varying by nation from 1:3 to 1:16 [31]. According to different research, the incidence of ASD is 1% in the global population, with males having a 4-6 times greater prevalence than females [32]. However, according to data from the US Centres for Disease Control and Prevention (CDC), up to 1/80 of children may be diagnosed with ASD, and over the past few years, diagnoses have significantly grown [8].

Studying alpha and beta diversities of microbiome in children with specific neurological disorders may contribute to identifying ways of addressing certain symptoms observed in these children [33]. More precisely, the measurement and constancy of the microbial composition of the cases (alpha diversity) and the differences in the microbial composition between individual cases (beta diversity) may promote the recognition of other putative pathophysiological characteristics of these neurological disorders and lead to changes in the treatment management plans [33]. The aim of the present systematic review is to identify and review evidence concerning the interaction between early exposure to the microbiome and neurodevelopmental disorders. The objectives of this review are as follows: This study aims to identify the kinds and types of microorganisms to which people are exposed early in life and examine the specific microbiome profiles associated with neurodevelopmental outcomes with an emphasis on understanding how the early-life microbiome may influence neurodevelopment. We also plan to assess the feasibility of manipulating early-life microbiomes to prevent or minimize neurodevelopmental issues, thus providing a systematic and scientific understanding of these associations.

## 2 METHODOLOGY

The study was performed as per the guidelines of Preferred Reporting Items for Systematic Review (PRISMA) as well as PROSPERO.

### 2.1 Study Participants

As per the microbes encountered during the early life of the participants in this review, they were selected. The age group of the participants was between 0-14 years. No restriction was placed on the gender or ethnicity of the participants. Only human studies have been included in the review.

### 2.2 Context and Outcomes

The purpose of this systematic review is to investigate the association between neurodevelopmental issues and the microbiome of early life. The principal outcomes comprise:

- The variety and kind of microorganisms that people are exposed to in their early years.
- The connection between neurodevelopmental processes and the makeup of the early-life microbiome.
- Evidence in favour of the theory that certain microbiome profiles in early life predispose people to neurodevelopmental issues.

### 2.3 Search Strategy

The review was carried out with the association of North South University, Dhaka, Bangladesh. The review utilized electronic searches of PubMed, Scopus, ISI Web of Science, and WHO Global Health Library (GHL). The search strategy included terms related to early life, microbiome, and neurodevelopmental disorders. Keywords used in the search were: ((early life) OR (infancy) OR (neonatal) OR (caesarean) OR (vaginal birth) OR (water birth)) AND ((microbiome) OR (microbiota) OR (microorganisms) OR (gut microbiome)) AND (((neurodevelopment) OR (neurological) OR (neurometabolic) OR (cognitive) OR (behavioral) OR (brain development)) AND ((disability) OR (disease) OR (outcome))) (Table **1**). The search strategy was last conducted on July 1, 2023. All citations identified were managed using EndNote software (v20.6). Bibliographies of relevant articles were also searched to ensure comprehensive coverage.

### 2.4 Eligibility Criteria

We used thorough inclusion and exclusion criteria in this systematic review to guarantee the calibre and applicability of the research taken into account. We considered original research publications without limitations on sex, race, ethnicity, or study design that explore the connection between the early-life microbiome and neurodevelopmental problems. The age group of this study was chosen between 0-14 years old. These articles covered investigations only on humans without taking into account animal or *in-vitro/in-vivo* studies. In particular, we chose English-language publications with adequate data for extraction and analysis. Studies with overlapping data sets, abstract-only publications, conference papers, letters, comments, theses, books, and author replies were not included. In order to preserve the integrity of the review, further papers with incomplete or faulty data were excluded, as well as those for which full text was not available. In order to find possibly relevant papers, at least two authors (TA and AH) independently evaluated the titles and abstracts first. Those that fit the requirements were then subjected to a full-text review. Inconsistencies in the selection of studies were settled by dialogue among all members of the review committee. This methodical methodology guarantees that the included researches are of high calibre and pertinent, offering solid proof of the impact of the early-life microbiome on neurodevelopmental outcomes. An agreement among the authors drove the final selection of research, ensuring that only those that satisfied all inclusion criteria were analysed and their data extracted. The trustworthiness of the review conclusions and the preservation of scientific rigour were contingent upon this stringent selection procedure.

### 2.5 Data Extraction

The abstracts and titles have been separately screened by authors (TA and AH). Additionally, the studies that did not meet the inclusion criteria were eliminated. After more debate, the writers addressed and resolved a number of issues. The same authors carried out the process of including and excluding research from full-text publications based on preset selection criteria. Using the data extraction sheet that was pilot tested, authors (TA and AH) separately extracted data in accordance with the study design. The study characteristics (*e.g*., authors, year of publication, region), participant details (*e.g*., age, sex, race, ethnicity), and details of the early-life microbiome exposure (*e.g*., type of microbiome, sources of exposure, maternal factors type, medical intervention) were all collected from each study using a standardised data extraction sheet. Meticulously gathered data on neurodevelopmental outcomes were centred on neurological, behavioural, and cognitive measurements. This methodical methodology guaranteed the systematic collection of all pertinent data.

### 2.6 Synthesizing Results

Using data from 19 suitable papers out of 2744 evaluated, the study conducted a systematic review and meta-analysis to synthesize results. In the synthesis, microbial groups (high/low presence) linked to neurodevelopmental disorders (ASD, ADHD) were extracted and compared. Using the Revman Web program, a meta-analysis was carried out with a random-effects model to take study variability into account. The total incidence of neurodevelopmental disorders was visualised using the forest plot model, which also provided effect sizes (odds ratios) and the degree of statistical heterogeneity (I^2^ = 97%). The considerable variation in research populations, techniques, and methods for microbiological examination served as justification for this model selection. Because of the considerable variability, higher sample sizes and standardised procedures are required for results that are more reliable. This methodology guarantees a thorough examination, using a range of research findings to clarify the relationship between early-life microbiome dysbiosis and neurodevelopmental disorders.

## 3 RESULTS

**Fig. (1):**
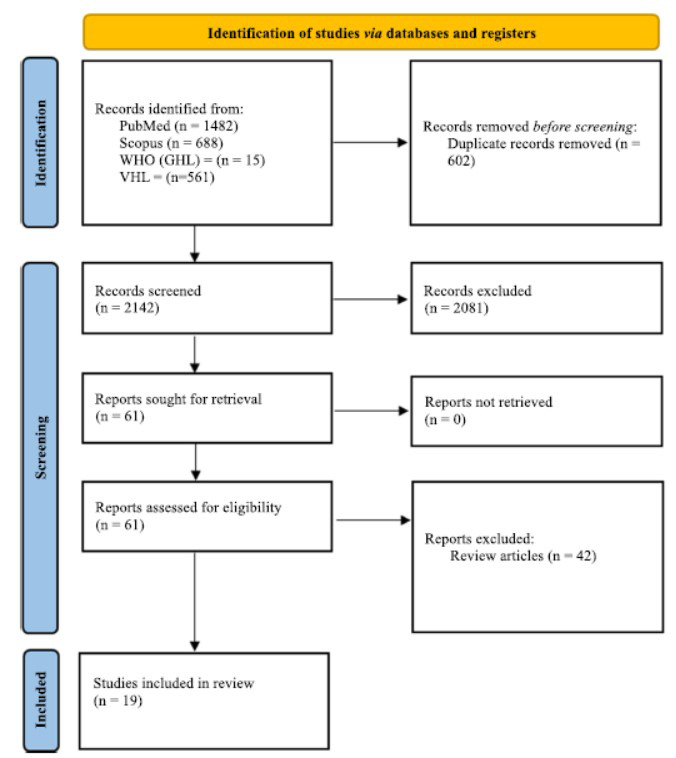
PRISMA flowchart of study selection process. After the identification of 2,744 studies, the subsequent steps of screening and eligibility were carried out. Finally, a total of 19 studies were finalized to be included.

Using database searches from PubMed, Scopus, WHO (GHL), and VHL, the systematic review identified 2,744 papers. A total of 602 studies were excluded after duplicate screening. Two thousand eighty-one studies were eliminated based on the second exclusion criterion, which included missing and irrelevant keywords and the inability to access the full text. After a thorough screening process, a total of 61 potential studies were reduced, and 19 of them (Table **2**) were included in our systematic review (Fig. **1**).

**Fig. (2):**
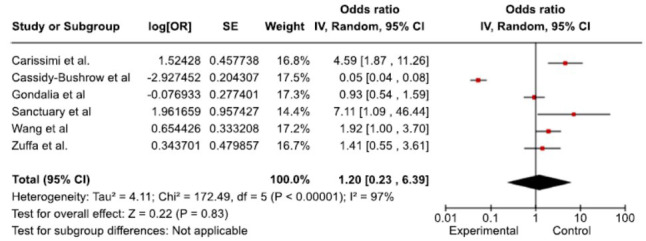
Forest plot for the overall prevalence of neurodevelopmental disorders among the study population. The figure indicates prevalence by random-effect meta-analysis with 6 studies. Here, the squares represent the effect sizes of the study, and the size of the squares shows the weight of the study.

The forest plot shows different results from six types of research looking at the prevalence of neurodevelopmental disorders among the study population (Fig. **2**). Significantly beneficial effects were observed by Carissimi *et al*. [48] and Sanctuary *et al*. [50] with ORs of 4.59 and 7.11, respectively. An OR of 0.05 indicated a substantial negative effect, as reported by Cassidy-Bushrow *et al*. [41] and Wang *et al*. [45], who discovered a marginally significant beneficial impact with an OR of 1.92, but Gondalia *et al*. [51] and Zuffa *et al*. [39] reported non-significant results with ORs of 0.93 and 1.41, respectively. With a high level of heterogeneity (I^2^ = 97%) indicating significant variability among the studies, the combined OR of 1.20 (0.23, 6.39) generally suggests no significant effect. This underscores the need for standardized methodologies, larger sample sizes, and further exploration to address heterogeneity and better understand the relationship being studied.

The abundance of different microbial groups and their correlation with neurodevelopmental disorders are shown in the table based on data from many research (Table **3**). While several Clostridium species are connected to ASD, high abundances of *Pasteurellaceae* and *Clostridiaceae* are associated with Down syndrome. In cases of ASD, low *Bifidobacterium* species abundances are also noted. There is a correlation between high *Dorea* and *Blautia* OTUs abundances and low *Enterococcus* and *Ruminococcus* abundances in ADHD. These results underline how gut bacteria could play a part in neurodevelopmental disorders.

### 3.1 Qualitative Assessment

Selected case-control studies on early-life microbiome exposure and neurodevelopmental outcomes were assessed using the Newcastle-Ottawa Quality Assessment Scale (NOS), which brought to light methodological strengths and flaws (Table **4**). Zuffa *et al*. [39] were able to secure the highest possible grade of seven out of nine stars because of their precise case definitions, representative samples, precise controls, strict control over confounders, regular exposure assessment, and handling of non-response rates. Following closely behind with six stars, Cassidy-Bushrow *et al*. [41] performed admirably in most categories but lacked comprehensive non-response rate control. Both Carissimi *et al*. [48] and Sanctuary *et al*. [50] received five stars for their strong methodology but with fewer confounders controlled and restrictions in their control definitions. Gondalia *et al*. [51] and Wang *et al*. [45] both earned four stars, suggesting considerable methodological rigour but serious inadequacies in exposure ascertainment, comparability, and control selection. Both Axelsson *et al*. [44] and Tamana *et al*. [35] received a grade of three stars, which was indicative of representative samples and well-defined case definitions but also of significant flaws in the control definition, comparability, and non-response rate management. While lower-scoring studies showed significant methodological gaps, highlighting the need for thorough control measures and detailed methodological documentation in future research, high-scoring studies demonstrated robust methodologies, offering trustworthy insights into the relationship between early-life microbiome exposure and neurodevelopmental outcomes.

## 4 DISCUSSION

**Fig. (3):**
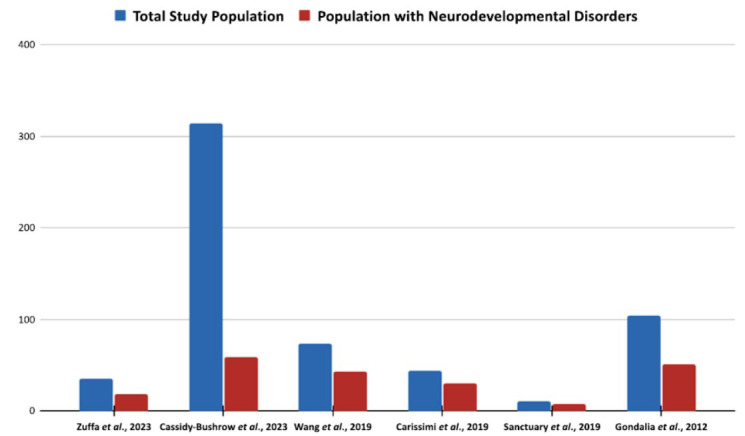
Comparison of total study population *vs*. population with neurodevelopmental disorders. In comparison to the group identified with neurodevelopmental disorders across many studies, the distribution of the entire study population is depicted in the picture. The overall number of study participants compared to those who were diagnosed with neurodevelopmental disorders is shown in the bar chart. For example, neurodevelopmental disorders were present in 59 (18.8%) out of 314 of the study population in Cassidy-Bushrow *et al*. [41], but Gondalia *et al*. [51] found 51 (49%) out of 104 study population to have neurodevelopmental disorders. This comparison helps uncover patterns and connections that may exist between early-life microbiome exposure and neurodevelopmental outcomes, as well as insight into the incidence of such diseases among the research cohorts.

The comparison of the total study population with the population with neurodevelopmental disorders in the identified society provides valuable information concerning the overall incidence and distribution of NDs. Neurodevelopmental disorders are the main factors that affect cognition and behaviour, including Attention-Deficit/Hyperactivity Disorder (ADHD) and Autism Spectrum Disorder (ASD). The goal of the present study was, therefore, to review the existing literature on any relationships between the early microbiome with these diseases in the selected study subjects. The analysis of the results of the studied research indicates that the incidence of neurodevelopmental issues differed in the investigated groups (Fig. **3**). For instance, Cassidy-Bushrow and colleagues’ study [41] had the largest sample of 314 individuals, of which 59 were diagnosed with neurodevelopmental disorders or approximately 18.8%. Similarly, Gondalia and colleagues [51] reported that 49% of them belonged to the group had neurodevelopmental disorders. These findings point out the severe impact that neurodevelopmental abnormalities have on the populations in question. Across the investigations, the proportion of favourable outcomes among the overall population was 54.3% for Zuffa *et al*. [39] and 72.7% for Sanctuary and colleagues [50]. These results show a high probability of neurodevelopmental disorders in these groups and suggest that there might be a correlation between the development of these disorders and early life exposure to the microbiome.

A better understanding of microbial abundance across different researches can also help in understanding the link between gut microbiota and neurological disorders. In a broader perspective of viewing these correlations or factors, it is possible to understand how variations in microbial presence impact diseases such as ADHD, DS, and ASD. Zuffa and colleagues revealed that *Clostridium clostridioforme*, *Clostridium neonatale*, *Clostridium difficile*, and *Clostridium bolteae* were among the *Clostridium* species which associated with ASD patients based on their study [39]. As per the MGBA theory [9, 10], gut microorganisms might have effects on brain function in a manner of ways including immunological, hormonal, and neurological pathways. The results which have emerged are in agreement with this theory. *Clostridium* species frequently colonize the gastrointestinal tract of autistic children due to their ability to produce neurotoxins and change intestinal permeability, which may affect normal brain development and worsen symptoms of ASD. It is additionally supported by the reduced amounts of *Bifidobacterium* species found in people with ASD; these good bacteria are crucial for preserving gut health and avoiding the colonisation of pathogens [25].

In the children with ADHD, Cassidy-Bushrow and colleagues [41] found that there are reduced *Enterococcus* and *Ruminococcus*, and increased *Dorea* and *Blautia* OTUs. There is also a possibility of disturbance of normal neurotransmitter pathways, such as dopamine and serotonin, that make up attention and behaviour regulation as a result of this microbial composition [8]. These microorganisms may also be involved in the modulation of neurodevelopment and the development of ADHD through the regulation of the production and metabolism of neurotransmitters [11]. Understanding the role of Firmicutes and Clostridia in neurodevelopment, as described in the studies by Carissimi *et al*. [48] and Wang *et al*. [45], is especially important given that these bacteria were found to be overrepresented in patients with ASD. *Firmicutes* seem to play a role in the production of SCFAs, which may affect behaviour and brain function through the MGBA [53]. Among SCFAs, butyrate has been shown to modulate neurotransmitter release, inflammation, and gene expression, which are fundamental to neurodevelopment [12]. These observations indicate a relationship between neurodevelopmental issues and dysbiosis in gut microbiota that is characterized by the high density of pathogenic bacteria and low density of beneficial microbes. Still, some secondary effects, which may cause neurodevelopmental issues, include altered intestinal permeability, shift in immunology reactions, and changes in neurotransmitter production.

**Fig. (4):**
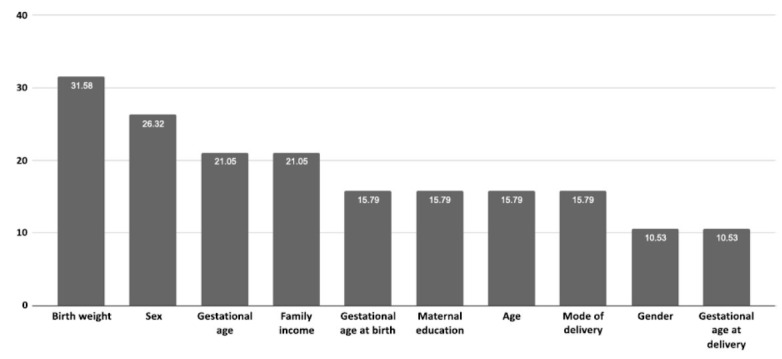
Percentage of covariates among studies. Covariates found in the studies looking at the relationship between early-life microbiome and neurodevelopmental disorders are shown in the chart. With 31.58% of the studies mentioning it, birth weight is the most commonly reported covariate. It is followed by sex (26.32%), gestational age (21.05%), and family income (21.05%). Gestational age at birth, maternal education, age, delivery method, gender, and gestational age at delivery are additional important factors.

Concerning the covariates, the findings revealed that birth weight is an essential sign of child health and its relationship with neurodevelopment (Fig. **4**). Delays in development and impairments in cognitive skills have been associated with low weight at birth [54]. This connection highlights the importance of monitoring birth weight and managing it to minimize potential negative effects. Sex is another covariate of the overall prevalence and symptom patterns of neurodevelopmental disorders. The research has found that boys are more likely to develop certain disorders, including ADHD and ASD [55]. Furthermore, it was also apparent that child development is one of the aggregately and this was in line with the socioeconomic status of the mother and the family income. That being said, limited access to healthcare, nutrition, and stimulating environments, all essential for optimal neurodevelopment, is linked to lower family income and mother education levels [56]. The evaluation of the factors revealed that the delivery mode (vaginal and caesarean) had an impact on the early microbial colonisation of the baby's gut. Alteration in the makeup of the microbiome following caesarean sections has been linked to immune system development and neurodevelopmental health [57]. When feasible, suggestions for delivery techniques can be informed by an understanding of these effects. Complex interactions, including genetic, epigenetic, and environmental variables, exist between these covariates and the early-life microbiome. The gut microbiota affects brain functioning through immunological, hormonal, and neurological pathways, and this microbiota-gut-brain axis is crucial for neurodevelopment [58]. This relationship is important for neurodevelopment. Neurodevelopmental outcomes can be affected by disruptions in the makeup of the gut microbiota, which are frequently observed in preterm babies or those born *via* caesarean section.

The suppression of butyrate levels is common in ASD [59] and ADHD [60]. This has a number of consequences for many of the wider aspects of ASD and ADHD pathophysiology. Butyrate is a metabolic substrate for intestinal epithelial cells [61] and astrocytes [62], two key cells in regulating alterations in the gut-brain axis [63]. In addition to providing energy for neuronal and epithelial cells, butyrate also maintains immunological homeostasis in the gut environment, which is crucial to its function in ASD and ADHD. Gut permeability may rise as a result of the loss of butyrate-producing bacteria, allowing pro-inflammatory chemicals to enter the bloodstream and perhaps influencing neurodevelopment. Decreased capacity to produce melatonin has been shown in ASD platelets, pinealocytes, and intestinal epithelial cells, driven by miR-451 suppression of 14-3-3 proteins [63]. As butyrate increases melatonin [61] and melatonin is an important aspect of ASD and ADHD pathophysiology [64, 65], including *via* the optimization of mitochondrial function [66], the suppression of gut microbiota that upregulate butyrate is important to wider ASD and ADHD pathophysiology. Butyrate's involvement in upregulating melatonin production is essential for neurodevelopmental disorders because melatonin has the ability to stabilise mitochondrial activity, which is frequently disturbed in these circumstances. Butyrate disruption impacts these intercellular connections by compromising vagal nerve signalling and enteric glial cell function, both of which are essential for gut-brain communication pathways. This may be of particular importance in the development of the intercellular interactions of the wider gut microenvironment, including enteric glial cells, the enteric nervous system, the mucosal immune system, and their interactions with the vagal nerve [67, 68]. Importantly, alterations in the early developmental gut microbiome will impact how the gut microbiome influences the development of the amygdala, and therefore with the integration of sensory and cognitive processes with affect/danger detection [67, 69]. Research indicates that butyrate and other SCFAs affect the development of the amygdala by regulating neurotransmitter production and inflammation, which may have an effect on how emotions and sensory information are processed in ASD [70]. Future research should clarify these interactions of the gut microbiome with wider developmental processes in other organs and tissues.

A notable limitation of the research is the restricted number of studies and their shared research objectives, which created a substantial obstacle to conducting an exhaustive analysis of the data. It's difficult to construct large, broadly applicable findings from an insignificant amount of research. Adding to the difficulty of integrating data into a coherent understanding is the diversity in research populations, approaches, and microbiological analysis methods across the included studies. Furthermore, most of the research used cross-sectional designs, which makes it more difficult to prove a link between the compositions of microbes and the results related to neurodevelopment. More solid proof of the temporal correlations and perhaps causal effects of the gut microbiota on neurodevelopment would come from longitudinal investigations. Another limitation pertains to the variability observed in the demographic attributes of research participants, encompassing variations in age groups, geographical locales, and socioeconomic statuses. It is challenging to directly compare the findings of different research because of the potential effect of these changes on the gut microbiota and neurodevelopmental outcomes.

## CONCLUSION

This study emphasises how early-life gut microbiota plays a critical role in the development of neurodevelopmental disorders. Different microbial profiles were shown to be linked to disorders, including ADHD and ASD. Numerous *Clostridium* species seemed to be common in ASD. The findings imply that these bacterial families may affect neurodevelopment through processes involving immune regulation and neuroinflammation. The study also laid stress on the need for *Bifidobacterium* and other helpful bacteria for the health of the gut and to avoid pathogenic colonisation. However, none have provided longitudinal data or had a huge number of participants to show that such links are causal in nature due to method variance, and a few studies on this. Nonetheless, current studies are limited by one or the other of these constraints, and therefore, larger, more diverse population samples, as well as more longitudinal designs, should be used in the future to better investigate the temporal aspects of interactions between microbiome and neurodevelopment.

## Figures and Tables

**Table 1:** Database search strategy summary.

**Database**	**Keywords**	**Total**
PubMed	((early life) OR (infancy) OR (neonatal) OR (caesarean) OR (vaginal birth) OR (water birth)) AND ((microbiome) OR (microbiota) OR (microorganisms) OR (gut microbiome) OR (gut microbiota)) AND (((neurodevelopment) OR (neurological) OR (neurometabolic) OR (cognitive) OR (behavioral) OR (brain development) OR (autism) OR (attention-deficit hyperactivity disorder)) AND ((disability) OR (disease) OR (outcome)))	1482
WHO (GHL)	((early life) OR (infancy) OR (neonatal) OR (caesarean) OR (vaginal birth) OR (water birth)) AND ((microbiome) OR (microbiota) OR (microorganisms) OR (gut microbiome) OR (gut microbiota)) AND (((neurodevelopment) OR (neurological) OR (neurometabolic) OR (cognitive) OR (behavioral) OR (brain development) OR (autism) OR (attention-deficit hyperactivity disorder)) AND ((disability) OR (disease) OR (outcome)))	15
VHL	((early life) OR (infancy) OR (neonatal) OR (caesarean) OR (vaginal birth) OR (water birth)) AND ((microbiome) OR (microbiota) OR (microorganisms) OR (gut microbiome) OR (gut microbiota)) AND (((neurodevelopment) OR (neurological) OR (neurometabolic) OR (cognitive) OR (behavioral) OR (brain development) OR (autism) OR (attention-deficit hyperactivity disorder)) AND ((disability) OR (disease) OR (outcome)))	561
Scopus	((early life) OR (infancy) OR (neonatal) OR (caesarean) OR (vaginal birth) OR (water birth)) AND ((microbiome) OR (microbiota) OR (microorganisms) OR (gut microbiome) OR (gut microbiota)) AND (((neurodevelopment) OR (neurological) OR (neurometabolic) OR (cognitive) OR (behavioral) OR (brain development) OR (autism) OR (attention-deficit hyperactivity disorder)) AND ((disability) OR (disease) OR (outcome)))	688
**Total**		2,746

**Table 2:** Summary of the studies.

**Author**	**Year Published**	**Study Population**	**Population with Neurodevelopmental Disorders**	**Neurodevelopmental Disorders Type**	**Age Group**	**List of Covariates**	**Microbiome**	**References**
Sordillo *et al.*	2019	309	N/A	Communication disorders, personal and social skills delays, and fine motor skills delays	3 years	Clinical site, mode of delivery, child's sex, antibiotic administration in the first days of life, gestational age at birth, maternal age, marital status, educational level, family income, and infant race/ethnicity.	*Lachnospiraceae* and unclassified *Clostridiales* taxa were associated with poorer communication and personal and social scores, while *Bacteroides* were associated with poorer fine motor scores.	[34]
Tamana *et al.*	2021	405	N/A	Autism spectrum disorders (ASD) and attention-deficit/ hyperactivity disorder (ADHD)	1-2 years	Gender, maternal ethnicity, family income, birth mode, direct antibiotic exposure, older sibling, ear infection, breastfeeding status at 6 months, maternal pre-pregnancy weight, gestational age at delivery, maternal prenatal fruit intake, and age at microbiota sampling.	*Bacteroidetes*-dominant and *Firmicutes*-dominant microbiota clusters	[35]
Gao *et al*.	2019	39	N/A	N/A	1 year	Income, maternal/paternal psychosis, gestational age at birth, postnatal age at scan, birth weight, stay in neonatal intensive care, APGAR scores, maternal/paternal age, maternal/paternal education, surgical anesthesia, older siblings, current breastfeeding status, formula feeding status, type of milk received other than breast milk or formula, symptoms of illness in the previous week, gastrointestinal symptoms in the previous week, antibiotics during the first year, and maternal use of antibiotics during pregnancy.	N/A	[36]
Tomlinson *et al*.	2019	807	N/A	Intellectual deficits, cognitive impairment, and language deficits​	10 years	Infant sex, gestational age, birth weight Z-score <−1, maternal education, antenatal steroid use, histologic inflammation of the chorion/decidua, and mother’s eligibility for government-provided medical care insurance.	*Ureaplasma urealyticum* was associated with deficits in language and mathematics, while *Lactobacillus* sp. was associated with a decreased risk of cognitive impairment and language deficits	[37]
Hursitoglu	2021	43	21	Down syndrome (DS)	34.08 ± 5.48 years	Age, gestational age, medications affecting gut microbiota composition, presence of acute and/or chronic infection or inflammation, and presence of any malignancies​.	*Clostridiaceae* and *Pasteurellaceae*	[38]
Zuffa *et al*.	2023	35	19	Autism spectrum disorder (ASD)	5 to 36 months	Antibiotic exposure, preterm birth, medical conditions, maternal age, family income, and parental education level.	Infants at elevated likelihood of ASD harbored less *Bifidobacterium* and more *Clostridium*-related species at 5 months of age. The specific microorganisms identified were *Bifidobacterium breve, Bifidobacterium bifidum, Bifidobacterium longum, Bifidobacterium kashiwanohense, Clostridium clostridia forme, Clostridium neonatale, Clostridioides difficile, Clostridium bolteae, Blautia producta*, *Ruminococcus gnavus*, and *Klebsiella variicola*. Additionally, infants with an elevated likelihood of ASD excreted lower amounts of fecal GABA at 5 months of age.	[39]
Sun *et al*.	2020	34	N/A	N/A	36-38 weeks	Gender, delivery type, birth weight, feeding type, premature rupture of membranes (PROM), score for Neonatal Acute Physiology-Perinatal Extension-II (SNAPPE-II), birth weight, and percentage of feeding with mother’s breast milk (%MBM).	*Bacteroides*, *Clostridium*, and *Veillonella*	[40]
Cassidy-Bushrow *et al.*	2023	314	59	Attention Deficit Hyperactivity Disorder (ADHD)	10 years	Exact age at stool sample collection, maternal body mass index, prenatal antifungal use, maternal smoking during pregnancy, child sex, mode of delivery, gestational age at delivery, parity, and breastfeeding status.	*Enterococcus* and *Ruminococcus* OTUs (depleted) and *Dorea* and *Blautia* OTUs (enriched)	[41]
Oliphant *et al.*	2021	58	N/A	Suboptimal head circumference growth trajectories (SHCGT) as an early marker for potential neurodevelopmental issues.	0-36 weeks	Delivery mode, antibiotics, enteral feeding, gestational age at birth, birth weight, sex, morbidity, length of NICU stay, and postmenstrual age.	Depletion in the abundance of *Bacteroidota* and *Lachnospiraceae*. Specifically, *Bacteroidota* and *Lachnospiraceae* were identified as potential biomarkers for appropriate head circumference growth trajectories (AHCGT)	[42]
Sun *et al*.	2023	116	N/A	The study focused on neurodevelopmental outcomes measured by the Ages and Stages Questionnaires (ASQ), specifically looking at developmental domains such as communication skills, fine motor skills, gross motor skills, problem-solving ability, and personal social skills​.	1-3 years	Recruitment site, education level, marital status, family income, gestational age, Vitamin D treatment, maternal health status, and children’s ASQ measures.	*Fusobacteriia* was associated with high fine motor skills in the maternal prenatal gut microbiota but became associated with low fine motor skills in the infant gut microbiota. The study also identified the significance of *Bacteroidota* and *Firmicutes* phyla in the gut microbiome influencing neurodevelopmental outcomes.	[43]
Axelsson *et al.*	2019	671,592	17,971	Attention Deficit Hyperactivity Disorder (ADHD)	2 years	Childhood antibiotics use, mode of delivery, maternal age at birth, parental age difference, parental education, maternal marital status, maternal smoking, infant sex, 5-minute Apgar score, instrument use at delivery, use of CPAP or ventilator, asphyxia, parental epilepsy, preeclampsia or hypertension, gestational diabetes, parity, induction of labor, induction of contractions, maternal antibiotics use during pregnancy, maternal infections during pregnancy, and parental ADHD history.	N/A	[44]
Wang *et al*.	2019	74	43	Autism Spectrum Disorder (ASD)	2-8 years	Sleep problems, retrogressive behavior, family history of psychiatric disorders, prolonged labor, and gastrointestinal (GI) problem.	**Lower in number -** *Toxoplasma gondii,* *Streptococcus mutans, Mycobacterium tuberculosis*, human herpesvirus 2, herpes simplex virus, hepatitis C virus, yellow fever virus, simian immunodeficiency virus, Guanarito virus. **Higher in number -***Listeria monocytogenes*	[45]
Senn *et al*.	2019	1074	N/A	N/A	2 years	Maternal age at conception, paternal age at conception, maternal education, paternal education, paternal full-time employment, the socio-economic index for the area, maternal smoking at preconception or during pregnancy, any maternal antibiotic use during pregnancy, maternal pet ownership, gestation age, birth weight, mode of birth, any breastfeeding, duration of breastfeeding, post-natal antibiotic use in the first 12 months, attended daycare center in the first 12 months, and infant sex.	N/A	[46]
Kelsey *et al.*	2021	63	N/A	N/A	9 days to 56 days	Any antibiotic treatments, prenatal antibiotics, antibiotics during labor and delivery, postpartum administration to mother, administered directly to the infant between delivery and study appointment, Apgar Score at 1^st^ and 5^th^ minute, birth length, birth weight, Bristol Stool Scale Score, breastfeeding, epidural, gestational age, sex, income, maternal depression, maternal education, number of siblings, hours between stool sample collection and freezing, lived with pets, Pitocin, race, and vaginal delivery.	*Bifidobacteria* and *Bacteroides*	[47]
Carissimi *et al.*	2019	44	30	Autism Spectrum Disorder (ASD)	2-5 years	Sex, age, gastrointestinal (GI) symptoms, antibiotic use, use of probiotics/prebiotics, clinical observation, neurologic examination, Griffiths Mental Development Scales (GMDS), Autistic Diagnostic Observation Schedule (ADOS-2), and the CHARGE Gastrointestinal History Questionnaire (GIH).	Decreased *E. coli*	[48]
Carlson *et al.*	2018	89	N/A	N/A	1-2 years	Breastfeeding at the time of sample collection, birth method, paternal ethnicity, presence of older siblings, and various alpha diversity measures.	High abundance of *Faecalibacterium,* *Bacteroides,* and *Ruminococcaceae*	[49]
Sanctuary *et al.*	2019	11	8	Autism Spectrum Disorder (ASD)	2-11 years	Age, sex, initial gastrointestinal symptoms (constipation, diarrhea, both), irritable bowel syndrome (IBS), vomiting, gas/bloating, picky eating, low baseline BMI, dietary intake, and supplement compliance​	Lower overall abundance of potentially beneficial taxa, such as *Bifidobacterium* and *Akkermansia*. The presence of *Clostridia* and an abnormal ratio of *Firmicutes* to *Bacteroidetes* were noted as common in children with ASD.	[50]
Gondalia *et al.*	2012	104	51	Autism Spectrum Disorder (ASD)	2-12 years	Current gastrointestinal (GI) dysfunction, sex, autism severity, history of GI dysfunction, medically diagnosed GI disorder, current oral antibiotic use, current probiotics use, feeding type until age 6 months, food allergy, and immunization status	The study found no significant difference in the bacterial composition of the fecal material between the autistic group and their neurotypical siblings. The study identified *Firmicutes*, *Bacteroidetes*, *Proteobacteria*, and *Verrucomicrobia* as the most abundant phyla in all samples.	[51]
Acosta *et al*.	2014	1715	N/A	N/A	24 monts	Age, sex, socioeconomic status, dietary intake, micronutrient levels, breastfeeding status, presence of enteric infections, incidence of diarrhea, gut inflammation, and vaccination status	Enteropathogen infections contribute to undernutrition, growth faltering, and cognitive impairments. Key pathogens included *Salmonella, Shigella, Vibrio, Yersinia, Aeromonas, Campylobacter, Plesiomonas, Escherichia coli, rotavirus, norovirus, adenovirus, astrovirus, Cryptosporidium, Giardia, Entamoeba histolytica, Ascaris, Trichuris, Strongyloides, Cyclospora*, *Isospora*, and hookworm.	[52]

**Table 3:** Comparative analysis of microbial presence in neurodevelopmental disorder among various studies.

**Study**	**Microbial Group**	**Presence**	**Neurodevelopmental Disorder**
Zuffa *et al*. [39]	*Clostridium clostridioforme*	High	Autism spectrum disorder (ASD)
	*Clostridium neonatale*	High	
	*Clostridioides difficile*	High	
	*Clostridium bolteae*	High	
	*Bifidobacterium breve*	Low	
	*Bifidobacterium bifidum*	Low	
	*Bifidobacterium longum*	Low	
	*Bifidobacterium kashiwanohense*	Low	
Cassidy-Bushrow *et al*. [41]	*Dorea* OTUs	High	Attention Deficit Hyperactivity Disorder (ADHD)
	*Blautia* OTUs	High	
	*Enterococcus* OTUs	Low	
	*Ruminococcus* OTUs	Low	
Carissimi *et al*. [48]	*E. coli*	Low	Autism Spectrum Disorder (ASD)
	*Clostridia*	High	
	*Firmicute*	High	
Wang *et al*. [45]	*Listeria monocytogenes*	High	Autism Spectrum Disorder (ASD)
	*Toxoplasma gondii*	Low	
	*Streptococcus mutans*	Low	
	*Mycobacterium tuberculosis*	Low	
	Human herpesvirus 2	Low	
	Herpes simplex virus	Low	
	Hepatitis C virus	Low	
	Yellow fever virus	Low	
	Simian immunodeficiency virus	Low	
	Guanarito virus	Low	Autism Spectrum Disorder (ASD)
Sanctuary *et al*. [50]	*Bifidobacterium*	Low	
	*Akkermansia*	Low	

**Table 4:** Newcastle-Ottawa Quality Assessment Scale for cohort and case-control studies (NOS).

**Study**	**Diagnosis**	**Is the Case Definition Adequate?**	**Representativeness of the Cases**	**Selection of Controls**	**Definition of Controls**	**Comparability of Cases and Controls on the Basis of the Design or Analysis**	**Ascertainment of Exposure**	**The Same Method of Ascertainment for Cases and Controls**	**Non-response Rate**	**Total Stars**
Tamana *et al*., (2021)	ASD ADHD	*	*	-	-	-	*	-	-	3
Zuffa *et al*., (2023)	ASD	*	*	*	*	-	*	*	*	7
Cassidy-Bushrow *et al*., (2023)	ADHD	*	*	*	*	-	*	*	-	6
Axelsson *et al*., (2019)	ADHD	*	*	*	-	-	-	-	-	3
Wang *et al*., (2019)	ASD	*	*	*	-	-	-	-	*	4
Carissimi *et al*., (2019)	ASD	*	*	*	*	*	-	-	-	5
Sanctuary *et al*., (2019)	ASD	*	*	*	-	-	*	*	-	5
Gondalia *et al*., (2012)	ASD	*	*	*	*	-	-	-	-	4

## LIST OF ABBREVIATIONS

ADHD: Attention Deficit Hyperactivity Disorder
ASD: Autism Spectrum Disorder
CDC: Centres for Disease Control and Prevention
CNS: Central Nervous System
MGBA: Microbiota-gut-brain Axis
NDs: Neurodevelopmental Disorders
NOS: Newcastle-Ottawa Quality Assessment Scale
PRISMA: Preferred Reporting Items for Systematic Review
SCFA: Short-chain Fatty Acid
WHO: World Health Organization

## SUPPLEMENTARY MATERIAL

PRISMA checklist is available as supplementary material on the publisher’s website along with the published article.
